# Identification of Proanthocyanidins from Litchi (*Litchi chinensis Sonn*.) Pulp by LC-ESI-Q-TOF-MS and Their Antioxidant Activity

**DOI:** 10.1371/journal.pone.0120480

**Published:** 2015-03-20

**Authors:** Qiang Lv, Fenglei Luo, Xiaoyong Zhao, Yu Liu, Guibing Hu, Chongde Sun, Xian Li, Kunsong Chen

**Affiliations:** 1 Laboratory of Fruit Quality Biology/The State Agriculture Ministry Laboratory of Horticultural Plant Growth, Development and Quality Improvement, Zhejiang University, Zijingang Campus, Hangzhou, PR China; 2 College of Life Sciences, Zhejiang University, Zijingang Campus, Hangzhou, PR China; 3 College of Horticulture, South China Agricultural University, Guangzhou, Guangdong, China; Kobe University, JAPAN

## Abstract

Content of total proanthocyanidins as well as total phenolics, flavonoids, antioxidant activities were evaluated for litchi (*Litchi chinensis* Sonn.) pulp of 32 cultivars. One cultivar, Hemaoli, showed the highest total proanthocyanidins and total phenolics, and DPPH or ABTS radical scavenging activities. ESI-MS and NMR analysis of the Hemaoli pulp crude extracts (HPCE) showed that procyandins composed of (*epi*)catechin unites with degree of polymerization (DP) of 2–6 were dominant proanthocyanidins in HPCE. After the HPCE was fractionated by a Sephadex LH-20 column, 32 procyanidins were identified by LC-ESI-Q-TOF-MS in litchi pulp for the first time. Quantification of individual procyanidin in HPCE indicated that epicatechin, procyanidin B2, procyanidin C1 and A-type procyanidin trimer were the main procyanidins. The radical scavenging activities of different fractions of HPCE as well as six procyanidins standards were evaluated by both DPPH and ABTS assays. HPCE fractions showed similar antioxidant activities with those of Vc and six individual procyanidins, the IC_50_ of which ranged from 1.88 ± 0.01 to 2.82 ± 0.10 μg/ml for DPPH assay, and from 1.52 ± 0.17 to 2.71 ± 0.15 μg/ml for ABTS assay. Such results indicate that litchi cultivars rich in proanthocyanidins are good resources of dietary antioxidants and have the potential to contribute to human health.

## Introduction

**Fig 1:**
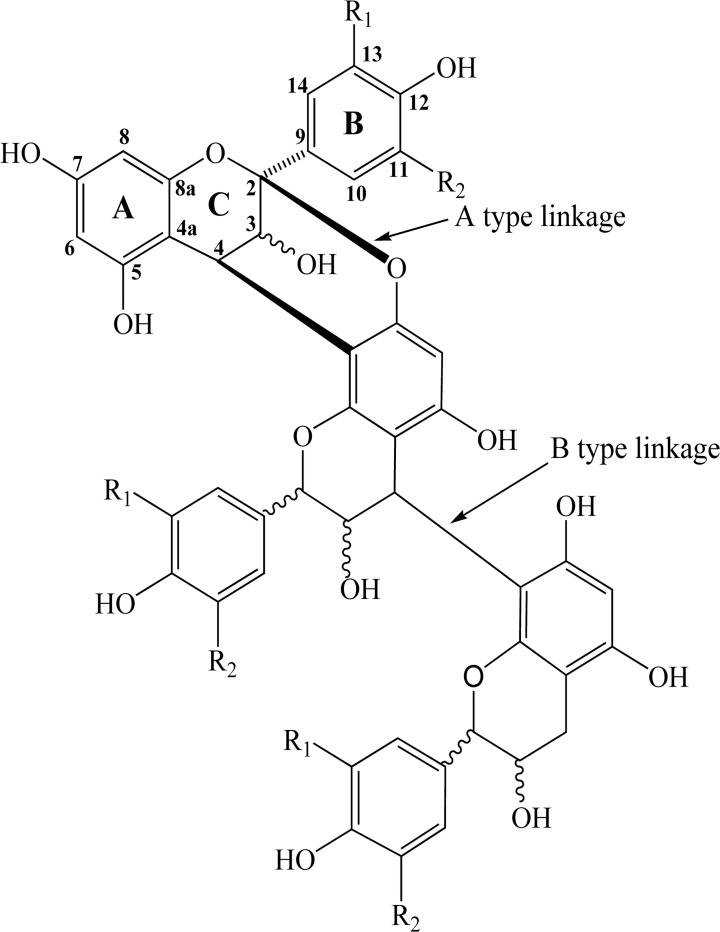
General structure of proanthocyanidins with A- and B-type linkage. (R1, R2 = H, propelargonidin; R1 = OH, R2 = H, procyanidin; R1, R2 = OH, prodelphinidin).

Proanthocyanidins (condensed tannins) are oligomers or polymers of flavan-3-ol units that are widely distributed in plants kingdom, e.g., apple, blueberry, chocolate, grape, and bark of pine [1–5]. Based on the hydroxylation patterns of their constitutive units, proanthocyanidins can be divided into several classes. Proanthocyanidins exclusively constituted of (*epi*)catechins units were procyandins, while proanthocyanidins consisted of (*epi*)afzelechin units and (*epi*)gallocatechins units were designated as propelargonidins and prodelphinidins, respectively. Procyanidins are the major proanthocyanidins in plants, while propelargonidins and prodelphinidins are less common [3, 6]. Flavan-3-ol units are usually linked via B-type bonds, i.e., C4→C8 or C4→C6 linkages (Fig. 1). However, additional C2→O7 or C2→O5 linkage leading to doubly bonded A-type proanthocyanidins may occur [7, 8]. Plant extracts rich in proanthocyanidins have been reported to have various bioactivities, such as antioxidant [9], anti-diabetic [10], anti-angiogenic [11], anti-carcinogenic [12], anti-inflammatory [13] and cardioprotective activities [14].

Litchi (*Litchi chinensis* Sonn.), a Sapindaceae plant native to southern China, is a rich source of natural phenolic compounds [15]. The pericarp [16, 17, 18], seed [19, 20] and flower [21] of litchi all contain significant amounts of procyandins. However, as the edible portion, litchi pulp has been rarely studied for their proanthocyanidin profiles. The present study was designed to investigate the proanthocyanidins in litchi pulp with HPLC-DAD, ESI-MS, ^13^C NMR, and LC-ESI-Q-TOF-MS. In addition, different litchi pulp extracts as well as six individual proanthocyanidin compounds were evaluated for their antioxidant activities.

## Materials and Methods

### Chemicals

(–)-Epicatechin, (+)-catechin, gallic acid, procyanidin A1, procyanidin A2, procyanidin B1, procyanidin B2, procyanidin C1, 2.2-diphenyl-1-picrylhydrazyl (DPPH), Folin-Ciocalteu reagent (2 mol/l), *p*-(Dimethylamino)cinnamaldehyde (DMAC), CD_3_OD and acetonitrile of chromatographic grade were purchased from Sigma-Aldrich (St. Louis, MO, USA). 2.2′-Azinobis (3-ethylbenzothiazoline-6-sulfonic acid) diammonium salt (ABTS) was purchased from Aladdin Inc (Shanghai, China). Double-distilled water (ddH_2_O) was used in all experiments and samples for HPLC-DAD and LC-ESI-Q-TOF-MS were filtered through a 0.22 μm membrane before injection. All the other reagents were of analytical grade and were bought from Sinopharm Chemical Reagent Co., Ltd. (Shanghai, China).

### Materials

Litchi fruit of 32 cultivars were harvested at commercial maturity in southern China in 2011 (Table 1). Among them, 17 cultivars were from Guangdong province, six cultivars from Guangxi province, five cultivars from Hainan province, three cultivars from Fujian province, and one cultivar from Yunnan province. They were either dominant commercial cultivars or endemic cultivars in different places of China (see S1 Table). All the litchi cultivars were acquired with permissions from their owners or preservers abiding by the laws in China. The plant materials used in this research did not involve endangered or protected species. The fruit were selected for uniformity of shape and color, and absence of disease and mechanical damage. The pulp was frozen in liquid nitrogen. After freeze-drying (FM 25EL-85, VirTis, USA), they were ground into a fine powder and stored at −80°C until extraction and analysis.

**Table 1:** Contents of total phenolics, total flavonoids, total proanthocyanidins, and antioxidant activities of pulp extracts of 32 litchi cultivars.

Cultivars	Total phenolics	Total flavonoids	Total proanthocyanidins	DPPH	ABTS
	(mg GAE/g DW)	(mg CE/g DW)	(mg PB2E/g DW)	(μg VcE/g DW)	(μg VcE/g DW)
Baila	4.59 ± 0.07	1.16 ± 0.03	1.28 ± 0.01	114.90 ± 2.57	126.71 ± 1.75
Baitangying	5.68 ± 0.14	1.92 ± 0.01	2.46 ± 0.01	130.32 ± 15.33	126.42 ± 3.35
Caomeili	4.51 ± 0.10	1.53 ± 0.02	1.77 ± 0.04	141.96 ± 2.40	135.23 ± 2.47
Chenzi	5.35 ± 0.08	1.06 ± 0.03	1.27 ± 0.04	151.18 ± 1.58	140.25 ± 1.99
Dadingxiang	5.68 ± 0.18	1.91 ± 0.03	2.09 ± 0.06	159.80 ± 3.27	151.90 ± 4.24
Dazao	7.27 ± 0.15	2.63 ± 0.01	3.16 ± 0.01	196.69 ± 2.82	174.91 ± 3.60
Feizixiao	8.50 ± 0.24	2.38 ± 0.04	2.68 ± 0.17	257.91 ± 5.89	211.18 ± 5.69
Guifeihong	4.14 ± 0.07	1.27 ± 0.02	1.53 ± 0.03	120.34 ± 5.38	122.73 ± 1.66
Guiwei	4.19 ± 0.07	1.39 ± 0.02	1.35 ± 0.08	100.69 ± 1.57	102.75 ± 1.62
Hemaoli	16.33 ± 0.02	6.09 ± 0.07	12.13 ± 0.02	367.37 ± 0.66	367.24 ± 0.50
Heiye	6.57 ± 0.32	2.16 ± 0.03	2.57 ± 0.01	177.79 ± 0.91	151.23 ± 7.62
Hexiachuan	6.86 ± 0.06	2.75 ± 0.02	3.27 ± 0.02	201.98 ± 2.98	170.74 ± 1.39
Huaizhi	4.94 ± 0.11	1.94 ± 0.07	2.08 ± 0.10	120.04 ± 3.16	120.08 ± 2.58
Hongxiuqiu	5.45 ± 0.17	2.38 ± 0.13	2.67 ± 0.10	186.26 ± 14.07	156.63 ± 4.12
Jianjianghongnuo	9.36 ± 0.01	4.20 ± 0.09	4.51 ± 0.17	228.74 ± 2.78	183.15 ± 0.16
Jinfeng	5.62 ± 0.04	2.35 ± 0.04	2.89 ± 0.05	163.58 ± 1.18	144.32 ± 0.90
Jinganghongnuo	5.55 ± 0.08	1.31 ± 0.03	1.54 ± 0.02	146.19 ± 2.23	138.92 ± 1.98
Jizuili	4.63 ± 0.14	1.73 ± 0.02	1.39 ± 0.06	125.78 ± 1.97	119.51 ± 3.28
Lanzhu	5.72 ± 0.13	1.56 ± 0.05	2.00 ± 0.04	133.19 ± 3.94	132.11 ± 3.20
Lingfengnuo	4.59 ± 0.12	1.90 ± 0.08	2.02 ± 0.07	156.17 ± 6.34	137.41 ± 2.97
Miaozhongnuo	3.95 ± 0.13	1.41 ± 0.04	1.59 ± 0.06	104.62 ± 3.12	104.36 ± 3.20
Mili	5.63 ± 0.04	1.09 ± 0.02	1.01 ± 0.01	107.04 ± 2.33	109.47 ± 1.07
Nuomici	3.91 ± 0.05	1.45 ± 0.03	1.55 ± 0.02	109.76 ± 1.59	104.17 ± 1.15
Qinzhouhongli	5.13 ± 0.17	1.71 ± 0.05	1.64 ± 0.07	130.17 ± 4.47	118.00 ± 4.03
Sanyuehong	7.19 ± 0.09	2.26 ± 0.05	2.77 ± 0.10	181.42 ± 2.05	158.53 ± 2.12
Shuangjianyuhebao	7.32 ± 0.05	2.04 ± 0.02	2.22 ± 0.05	212.56 ± 0.84	179.45 ± 1.28
Shuidong	9.71 ± 0.05	3.58 ± 0.05	3.63 ± 0.10	302.66 ± 2.94	182.10 ± 1.19
Wuheli	6.99 ± 0.07	2.62 ± 0.07	3.25 ± 0.09	216.34 ± 3.88	177.75 ± 1.62
Xianpoguo	4.73 ± 0.20	1.62 ± 0.04	1.84 ± 0.06	122.91 ± 2.53	118.19 ± 4.88
Yingshanhong	4.93 ± 0.09	1.57 ± 0.02	1.76 ± 0.04	129.86 ± 1.06	126.42 ± 2.23
Yuanhong	8.18 ± 0.01	2.58 ± 0.02	3.22 ± 0.07	230.85 ± 0.45	184.00 ± 0.16
Ziniangxi	4.73 ± 0.05	1.19 ± 0.03	1.30 ± 0.06	122.00 ± 1.64	116.86 ± 1.27

Data were presented as the mean ± S.D. (n = 3) on a dry weight (DW). Total phenolics were calculated as mg gallic acid equivalent (mg GAE/g DW). Total flavonoids were calculated as mg catechin equivalent (mg CE/g DW). Total procyanidins were calculated as mg procyanidin B2 equivalent (mg PB2E/g DW). The scavenging of DPPH and ABTS radicals were calculated as μg vitamin C equivalent (μg VcE/g DW), respectively.

### Preparation of pulp extracts of different litchi cultivars

One gram of lyophilized litchi pulp powder of each cultivar was extracted with 8 ml of 70% aqueous methanol by sonication for 30 min. The ultrasonic frequency and power were 60 kHz and 30 W, respectively. The extracts were centrifuged at 8000 rpm for 10 min at 4°C and the residue was extracted twice as above. All the supernatants were combined and used for determination of total phenolics, total flavonoids, total proanthocyanidins content and the antioxidant activity of the litchi fruit of different cultivars.

### Determination of contents of total phenolics, total flavonoids, and total proanthocyanidins in different litchi cultivars

Total phenolics of fruit extracts were measured using a modified colorimetric Folin-Ciocalteu method [22]. Four milliliters of ddH_2_O and 0.5 ml of appropriately diluted fruit extracts were placed in a test tube. Folin-Ciocalteu reagent (0.5 mol/l, 0.5 ml) was added to the solution and allowed to react for 3 min. The reaction was then neutralised with 1 ml of saturated sodium carbonate. Absorbance at 760 nm was measured using a spectrophotometer (Shimadzu, UV-2550) after 2 h. Gallic acid was used as the standard and data were expressed as mg gallic acid equivalents (GAE)/ g DW.

Total flavonoids of fruit extracts were measured according to a previous report [23] with some modification. One milliliter of ddH_2_O and 0.5 ml of appropriately diluted fruit extracts were placed in a test tube. Sodium nitrite (5%, 75 μl) was added to the solution and allowed to react for 6 min before adding 150 μl of aluminium chloride (10%). After 5 min, 0.5 ml sodium hydroxide (1 mol/l) was added and the final volume was adjusted to 2.5 ml with ddH_2_O. Absorbance at 510 nm was record immediately. (+)-Catechin was used as standard and data were expressed as mg catechin equivalents (CE)/ g DW.

Total proanthocyanidins of fruit extracts were measured according to a previous report [24] with modifications. DMAC solution (1 mg/ml) was prepared freshly with hydrochloric acid and ethanol (1:9, v/v). Appropriately diluted fruit extracts (50 μl) was added to 250 μl DMAC solution to initiate the reaction. After mixing, absorbance at 640 nm was recorded immediately by a microplate reader (Thermo, Electro Co., Waltham, USA). The content of total proanthocyanidins was expressed as mg procyanidin B2 equivalents (PB2E)/g DW.

### Fractionation of proanthocyanidins from Hemaoli pulp

Lyophilized Hemaoli pulp powder (300 g) was extracted with 2400 ml of 70% aqueous methanol by sonication for 30 min. The ultrasonic frequency and power were 60 kHz and 30 W, respectively. The extracts were filtered through Whatman No.1 paper, and the residue was extracted twice as above. All the supernatants were combined and evaporated by a rotary evaporator under reduced pressure at 45°C to remove methanol. Samples were then loaded to an Oasis HLB column (20 cc/1 g, Waters, Milford, MA, USA) to remove polysaccharides before elution with methanol. The eluent was then evaporated by a rotary evaporator to obtain the Hemaoli pulp crude extracts (HPCE) (3.17 g). This powder was used for ESI-MS and ^13^C NMR analysis (Bruker Avance III 600 NMR Instruments, Switzerland) for a preliminary identification of proanthocyanidins.

The HPCE were then dissolved in 10 ml 50% aqueous methanol and subjected to a Sephadex LH-20 column (16×400 mm) for fractionation. The absorbed litchi phenolics were eluted with gradient aqueous methanol from 0% to 60% in increments of 10% after each 900 ml elution volume. The fractions were collected and numbered from fraction #1 to 7. The column was then completely eluted with 900 ml of 100% methanol, which was collected as fraction #8. Each fraction was evaporated, which resulted in fraction 1 (F1, 0.31 g), fraction 2 (F2, 0.10 g), fraction 3 (F3, 0.06 g), fraction 4 (F4, 0.07 g), fraction 5 (F5, 0.14 g), fraction 6 (F6, 0.12 g), fraction 7 (F7, 0.21 g), and fraction 8 (F8, 1.34 g). These fractions were used for identification of proanthocyanidins and the antioxidant assays.

### HPLC-DAD analysis

Individual phenolic compounds in HPCE and different fractions of HPCE were analyzed by HPLC (2695 pump, 2996 diode array detector, Waters) coupled with an ODS C18 analytical column (4.6 × 250 mm), as previously described [25] with some modification. The column was operated at a temperature of 25°C. The compounds were detected between 200 and 400 nm. The mobile phase of HPLC consisted of 2% (v/v) acetic acid in water (eluent A) and of 0.5% acetic acid in water and acetonitrile (50:50, v/v; eluent B). The gradient program was as follows: 0–10 min, 5–13% of B; 10–15 min, 13–16% of B; 15–40 min, 16–40% of B; 40–43 min, 40–43% of B; 43–48 min, 43–50% of B; 48–58 min, 50–100% of B; 58–63 min, 100–5% of B.

### ESI-MS

Mass spectrometric analysis of HPCE were performed by an Agilent 6460 triple quadrupole mass spectrometer equipped with an ESI source (Agilent Technologies, USA) in negative ionization mode. The nebulizer pressure was set to 45 psi and the flow rate of drying gas was 5 l/min. The flow rate and the temperature of the sheath gas were 11 l/min and 350°C, respectively. The mass range was from *m/z* 50 to 2000. Chromatographic separations were done on an ODS C18 analytical column (4.6 × 250 mm) using an Agilent 1290 Infinity HPLC system (Agilent Technologies, USA). The eluent was split and approximately 0.3 ml/min was introduced into the mass detector. Quantification of the individual procyanidins were calculated as procyanidin B2 equivalent (PB2E), the selective ion monitoring (SIM) mode was used to select the molecular ions of the isomers from the procyanidins groups in litchi pulp extract for their quantification. An Agilent Mass Hunter Workstation was used for data acquisition and processing.

### NMR Spectroscopy

^13^C NMR spectra of the HPCE were recorded on a Bruker Avance III 600 NMR spectrometer (Switzerland) with CD_3_OD as solvent and tetramethylsilane as internal standard. (–)-Epicatechin, procyanidin A1, A2, B1, B2, and C1 were analyzed as standards.

### LC-ESI-Q-TOF-MS

The high resolution MS analysis of different fractions of HPCE was carried out by a Waters UPLC (Waters Corp., Milford, MA, USA) equipped with an AB Triple TOF 5600plus System (AB SCIEX, Framingham, MA, USA). The optimal MS conditions were as follows: the scan range was set at *m/z* 100–2000; the source voltage was −4.5 kV and the source temperature was 500°C in negative ionization mode; the pressure of Gas 1 (N_2_) and Gas 2 (N_2_) were set to 50 psi; and the curtain gas was set to 30 psi. For MS/MS, collision energy was −35 V; collision energy spread was 10 V; declustering potential was −100 V. The injection volume was set at 10 μl, and the UV detector was set at 280 nm. Maximum allowed error was set to ± 5 ppm. Chromatographic separations were done on an ODS C18 analytical column (4.6 × 250 mm) with 2% (v/v) acetic acid in water (eluent A) and 0.5% acetic acid in water and acetonitrile (50:50, v/v; eluent B) running under the same conditions as HPLC-DAD analysis. The eluent was split and approximately 1 ml/min was introduced into the mass detector. MS data were acquired during 0–63 min. Analyst^®^ TF 1.6 software (AB-Sciex) was used for data acquisition and processing.

### DPPH radical scavenging activity

DPPH radical scavenging activity was measured according to a previous report [26] with modifications. The reaction for scavenging DPPH radicals was carried out by adding 2 μl sample to 198 μl 25 μg/ml DPPH solution at 25°C. After 60 min, absorbance at 517 nm before (A_0_) and after (A_1_) the reaction was recorded by a microplate reader. DPPH radical scavenging activity of litchi pulp extracts of 32 cultivars were expressed as μg Vc equivalents (VcE)/g DW. For the radical scavenging activities of HPCE fractions and individual proanthocyanidins, IC_50_ values were calculated as the concentrations (μg/ml) that inhibited 50% of the DPPH radicals in the reaction, where radical scavenging activity was calculated as: Scavenging rate (%) = (A_0_ – A_1_)/A_0_ × 100%.

### ABTS radical scavenging activity

ABTS assay was carried out using a spectrophotometer as previously described [27]. ABTS radical cation was generated by reacting 7 mmol/l ABTS with 2.45 mmol/l potassium persulfate, and the mixture was allowed to stand in the dark at 25°C for 16 h before use. The ABTS solution was diluted with ethanol to an absorbance of 0.70 ± 0.05 at a wavelength of 734 nm before analysis. After mixing of 0.1 ml of the tested samples with 3.9 ml of ABTS solution, the absorbance at 734 nm was recorded for 6 min. ABTS radical scavenging activity of litchi pulp extracts of 32 cultivars were expressed as μg Vc equivalents (VcE)/g DW. For the DPPH and ABTS radical scavenging activities of HPCE fractions and individual proanthocyanidins, IC_50_ values were calculated as the concentrations (μg/ml) that inhibited 50% of the ABTS radicals in the reaction, where radical scavenging activity was calculated as: Scavenging rate (%) = (A_0_ – A_1_)/A_0_ × 100%.

### Statistical analysis

Experiments were performed in triplicate and data were expressed as the mean ± standard deviation. OriginPro 8.0 software packages (Originlab Corporation, Northampton, MA, USA) was used for statistical analysis of the experimental data.

## Result and Discussion

### Total phenolics, total flavonoids, total proanthocyanidins content in litchi pulp extracts of 32 cultivars

Plant phenolics constitute one of the major groups of compounds acting as primary antioxidants or free radical terminators in fruits, vegetables, or medicinal plants [28, 29, 30]. Phenolic content in plant depends on both intrinsic (genetic) and extrinsic (agronomic, environmental, postharvest handling and storage) factors. In the present study, total phenolics, total flavonoids content as well as total proanthocyanidins were analyzed for litchi pulp of 32 cultivars. Significant differences in the contents of these phenolics were observed among different cultivars (Table 1). Hemaoli, a cultivar planted mainly in Yunnan province, China, showed the highest total phenolics content (16.33 ± 0.02 mg GAE/g DW), total flavonoids content (6.09 ± 0.07 mg CE/g DW) and total proanthocyanidins (12.13 ± 0.02 mg PB2E/g DW). Furthermore, it also showed the highest antioxidant activity based on both DPPH (367.37 ± 0.66 μg VcE/g DW) and ABTS (367.24 ± 0.50 μg VcE/g DW) radical scavenging activities. Besides Hemaoli, the total phenolics contents of other cultivars ranged from 3.91 ± 0.05 to 9.71 ± 0.05 mg GAE/g DW, total flavonoids content from 1.06 ± 0.03 to 4.20 ± 0.09 mg CE/g DW, and total proanthocyanidins from 1.01 ± 0.03 to 4.51 ± 0.17 mg PB2E/g DW, the DPPH radical scavenging activities from 100.69 ± 1.57 to 302.66 ± 2.94 μg VcE/g DW and ABTS radical scavenging activities from 102.75 ± 1.62 to 211.18 ± 5.69 μg VcE/g DW. Therefore, Hemaoli pulp was chosen for further fractionation and identification analysis.

### Preliminary identification of proanthocyanidins in HPCE by ESI-MS

**Fig 2:**
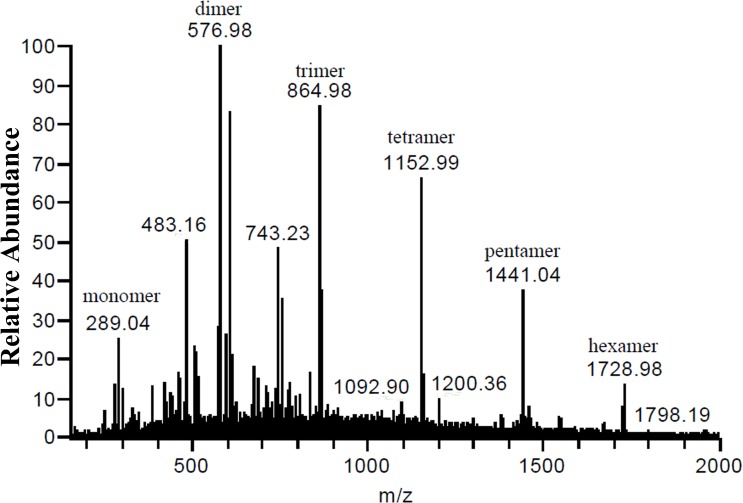
Direct infusion ESI-MS spectrum of Hemaoli pulp crude extracts (HPCE) in the negative mode.

ESI-MS spectra of the HPCE showed a series of polyflavan-3-ols (Fig. 2). The [M-H] ^−^ ion at *m/z* 289.04 suggested the molecular weight of 290 of (*epi*)catechin. The addition of molecular weight of 288 resulted in series abundant ions with *m/z* 576.98, 864.98, 1152.99, 1441.04, and 1728.98, corresponding to the molecular masses of procyanidins with the degree of polymerizations (DPs) of 2–6 (Fig. 2). Such results revealed that the dominant proanthocyanidins in HPCE were procyandins with relative low DPs. Procyanidins composed of (*epi*)catechin were found as predominant proanthocyanidins in the pericarp [18] and seed [19, 20] of litchi, though some prodelphinidins such as (–)-gallocatechin and (–)-epicatechin-3-gallate were also observed in the seed [19]. Higher DPs up to 22 and 20 were exhibited in litchi pericarp and seed, respectively [16, 31]. The DP of proanthocyanidins varies greatly with different plant tissues [32, 33, 34]. High DPs of proanthocyanidins up to 25, 14, 10 were observed in pear juice [35], pine bark [5], and mangosteen pericarps [32], respectively. The average DPs of proanthocyanidins were also studied in different plant tissues, where litchi pericarp showed an average DP of 6.4 [16], mangosteen pericarp showed an average DP of 6.6 [32], and grape seed, blueberry and green pear showed average DPs of 16.1, 14.0 and 10.3, respectively [36]. Proanthocyanidins with lower DP were more easy absorbed *in vivo* than those with higher DPs [37, 38], indicating that litchi pulp procyandins with DPs of 2–6 may have better bioavailability than those in litchi seed and pericarp.

### Identification of proanthocyanidins in HPCE by ^13^C NMR

**Fig 3:**
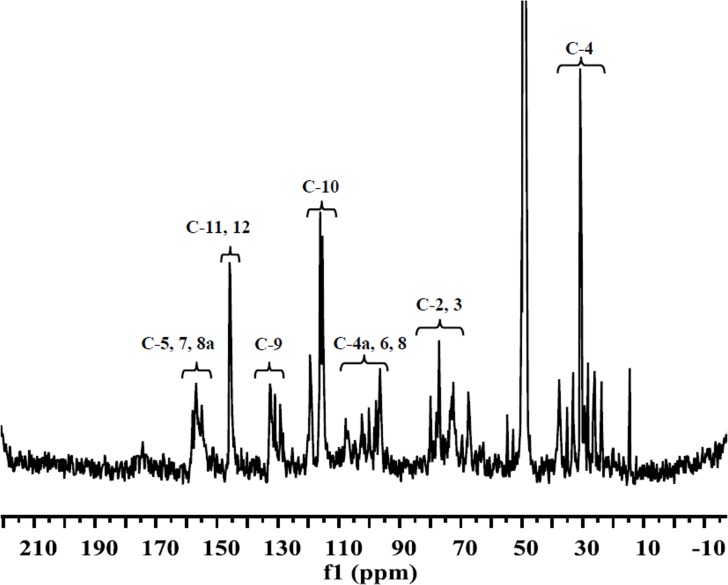
^13^C NMR spectrum of HPCE. Samples were dissolved in CD_3_OD.

^13^C NMR was used to obtain additional evidence for the proanthocyanidins of procyanidin type. The ^13^C NMR spectra of HPCE powder showed characteristic peaks for the A-ring carbons (150–160 ppm, 96–108 ppm), B-ring carbons (116–119 ppm, 130–133 ppm) and C-ring carbons (20–38 ppm, 67–100 ppm) of procyanidins (Fig. 3), which were consistent to the ^13^C NMR signals from standards of (–)-epicatechin and its oligmers (Table 2). There were also some A type linkages indicated from the signals at 152 ppm due to C7 of the A ring involved in the double linkage and the chemical shift of the C2 formed as a result of this additional bond observed at 100 ppm (Fig. 3). Such observation was consistent with previous report [32].

**Table 2:** ^13^C (150 MHz) NMR data of (–)-epicatechin, procyanidin A1, A2, B1, B2, and C1 in CD_3_OD.

Position	(–)-Epicatechin	Procyanidin A1		Procyanidin A2		Procyanidin B1		Procyanidin B2		Procyanidin C1		
		Upper unit	Terminal unit	Upper unit	Terminal unit	Upper unit	Terminal unit	Upper unit	Terminal unit	Upper unit	Middle unit	Terminal unit
C-2	79.9	100.4	84.6	100.2	81.8	82.4	77.2	79.8	77.2	79.8	77.1	77.1
C-3	67.5	67.9	68.2	68.2	67.1	68.6	73.3	67.1	73.6	66.9	73.0	73.5
C-4	29.3	29.3	29.1	29.3	30.0	20.9	37.2	29.8	37.2	30.8	37.5	37.4
C-4a	100.1	104.1	103.2	104.3	102.5	97.1	101.4	100.6	101.5	100.7	102.1	101.5
C-5	158.1	156.8	156.2	158.2	156.7	155.7	157.8	156.6	157.9	156.9	156.6	158.4
C-6	96.5	98.2	96.6	98.4	96.6	96.4	96.0	97.5	96.6	97.7	97.6	96.3
C-7	157.7	158.2	152.3	158.2	152.4	156.2	157.8	156.6	158.6	156.9	157.3	158.6
C-8	96.0	96.6	106.9	96.6	107.3	107.9	93.3	107.4	96.2	107.7	107.3	96.7
C-8a	158.1	154.5	151.5	154.3	152.2	154.0	158.5	154.7	158.2	154.6	155.0	158.0
C-9	132.4	132.4	130.6	132.5	131.3	132.5	132.9	132.2	132.7	132.2	132.6	131.8
C-10	115.4	115.7	115.8	115.6	116.0	114.9	115.4	115.4	115.4	115.4	115.2	115.4
C-11	145.8	146.9	146.9	145.7	146.1	146.0	145.6	145.9	145.9	146.0	145.9	145.6
C-12	146.0	146.4	146.4	146.8	146.4	145.9	145.9	145.7	145.7	145.6	145.5	145.5
C-13	115.9	116.4	115.8	115.7	116.1	116.2	116.0	116.0	116.0	116.1	116.1	116.1
C-14	119.5	119.9	120.8	119.8	120.4	119.5	119.5	119.2	119.4	119.2	118.9	119.4

### Identification of individual procyanidins by LC-ESI-Q-TOF-MS

**Fig 4:**
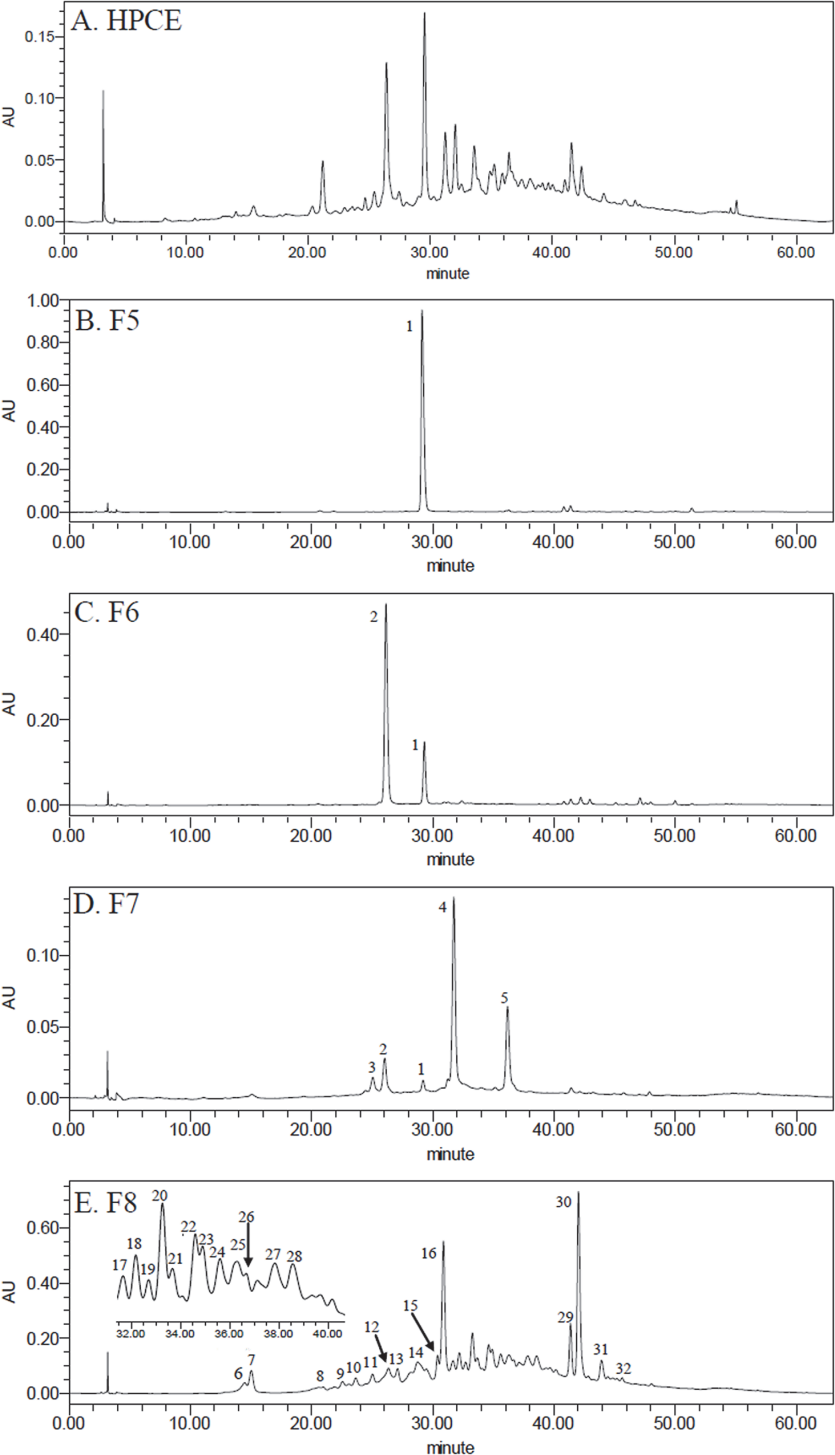
HPLC (280 nm) analysis of HPCE and its different fractions. A: HPCE; B: The fraction eluted with 40% methanol, F5; C: The fraction eluted with 50% methanol, F6; D: The fraction eluted with 60% methanol, F7; E: The fraction eluted with 100% methanol, F8.

Based on different molecular masses, proanthocyanidins can be separated on a Sephadex LH-20 column [39, 40]. In the present study, elution of sephadex column with increasing concentrations of methanol (0%-60%, and 100%) resulted in better separation of the main procyanidins in HPCE (Fig. 4). Fractions eluted with 40% methanol (Fig. 4B, F5), 50% methanol (Fig. 4C, F6), 60% methanol (Fig. 4D, F7), and 100% methanol (Fig. 4E, F8) contained the majority of procyanidins in HPCE (Fig. 4A). Therefore, these four fractions (F5, F6, F7, F8) were further analyzed by LC-ESI-Q-TOF-MS respectively.

In F5, peak 1 resulted in [M-H] ^−^ ion at *m/z* 289.0718, suggesting the molecular weight of 290 of (*epi*)catechin (Fig. 4B and Table 3). It was confirmed as (–)-epicatechin with the literature and chemical standard [41].

**Table 3:** Tentative identification and quantification of procyanidins in HPCE by using LC-ESI-MS.

Peak no.	R_t_	HPLC-DAD	Molecular	[M-H]^−^	[M-H]^−^	MS^2^	Tentative identification	ppm	Content
	(min)		weight	Obs	Cal				(mg PB2E/100 g DW)
1	29.13	231.0, 279.4	290	289.0718	289.0725	289.0728, 245.0825, 151.0403	(–)-Epicatechin	2.6	9.88±0.91
2	26.13	233.3, 279.4	578	577.1352	577.1348	577.1387, 451.1054, 425.0889, 407.0779, 289.0716, 245.0808	Procyanidin B2	-0.6	12.25±0.71
3	25.05	232.2, 279.4	866	865.1985	865.1991	739.1730, 577.1383, 451.1044, 407.0781, 287.0564, 245.0454	B-type procyanidin trimer	0.6	0.14±0.01
4	31.73	231.0, 279.4	866	865.1985	865.1992	739.1722, 577.1378, 451.1054, 407.0793, 287.0575, 245.0460	Procyanidin C1	-0.8	1.62±0.11
5	36.17	229.8, 278.2	864	863.1829	863.1843	863.1920, 711.1418, 693.1312, 575.1228, 423.0743, 285.0412	A-type procyanidin trimer	1.6	0.17±0.01
6	14.48	227.4, 278.2	1152	1151.2463	1151.2464	1151.2625, 863.1930, 711.1430, 573.1079	A-type procyanidin tetramer	0.1	0.02±0.00
7	15.03	231.0, 279.4	1152	1151.2463	1151.2474	1151.2573, 863.1906, 711.1403, 573.1068, 451.1054	A-type procyanidin tetramer	1.0	0.07±0.00
8	20.97	229.8, 279.4	1440	1439.3097	1439.3082	N.D.	Procyanidin pentamer	-1.0	0.02±0.00
9	22.56	231.0, 279.4	1440	1439.3097	1439.3085	N.D.	Procyanidin pentamer	-0.8	0.03±0.00
10	23.65	229.8, 278.2	866	865.1987	865.1988	865.2075, 577.1381, 449.0889, 287.0558, 245.0445	B-type procyanidin trimer	0.3	0.23±0.02
11	25.03	231.0, 279.4	866	865.1987	865.1987	865.2073, 577.1379, 451.1037, 287.0556, 245.0444	B-type procyanidin trimer	0.2	0.08±0.00
12	26.35	231.0, 279.4	578	577.1352	577.1344	577.1389, 451.1054, 425.0895, 407.0785, 289.07232, 245.0816	B-type procyanidin dimer	-1.3	0.12±0.01
13	27.10	231.0, 279.4	1154	1153.2679	1153.2609	1153.2732, 863.1915, 575.1230, 287.0562	B-type procyanidin tetramer	-0.9	0.04±0.00
14	28.76	232.0, 279.4	864	863.1829	863.1838	863.1932, 711.1439, 573.1091, 451.1065, 411.0749, 289.0733	A-type procyanidin trimer	1.1	0.27±0.01
15	30.40	231.0, 279.4	864	863.1829	863.1836	863.1918, 711.1423, 573.1075, 451.1050, 411.0734, 289.0722	A-type procyanidin trimer	0.9	0.31±0.01
16	30.88	235.7, 279.4	864	863.1829	863.1835	863.1908, 711.1411, 573.1072, 451.1048, 41.0730, 289.0721	A-type procyanidin trimer	0.8	1.21±0.11
17	31.67	228.6, 279.4	866	865.1985	865.1998	865.2071, 739.1740, 577.1391, 451.1054, 287.0566	B-type procyanidin trimer	1.5	0.09±0.00
18	32.19	231.0, 278.2	1152	1151.2463	1151.2494	1151.2599, 861.1776, 739.1747, 577.1393	A-type procyanidin tetramer	2.7	0.10±0.00
19	32.71	228.6, 279.4	1154	1153.2619	1153.2623	1153.2759, 863.1917, 575.1238, 287.0567	B-type procyanidin tetramer	0.3	0.08±0.00
20	33.27	232.2, 279.4	1154	1153.2619	1153.2620	1153.2734, 863.1917, 575.1227, 287.0561	B-type procyanidin tetramer	0.1	0.50±0.06
21	33.67	231.0, 279.4	866	865.1985	865.1986	865.2071, 739.1729, 577.1385, 451.1053, 287.0566	B-type procyanidin trimer	0.1	0.08±0.00
22	34.61	229.8, 279.4	866	865.1985	865.1991	865.2078, 739.1736, 577.1376, 575.1230, 451.1040, 287.0556	B-type procyanidin trimer	0.6	0.11±0.00
23	34.90	231.0, 279.4	1154	1153.2619	1153.2614	1153.2757, 865.2075, 575.1227, 413.0892, 287.0569	B-type procyanidin tetramer	-0.5	0.13±0.01
24	35.61	231.0, 279.4	864	863.1892	863.1837	863.1941, 711.1424, 693.1319, 575.1239, 451.1056, 289.0719	A-type procyanidin trimer	0.9	0.14±0.00
25	36.299	228.6, 279.4	866	865.2019	864.2020	865.2023, 577.1338, 407.0781, 287.0560, 243.0292	A-type procyanidin trimer	0.1	0.09±0.01
26	36.671	231.0, 279.4	1152	1151.2463	1151.2463	1151.2593, 861.1768, 739.1736, 577.1376, 411.0734, 287.0560	A-type procyanidin tetramer	0	0.04±0.00
27	37.83	231.0, 279.4	1152	1151.2440	1151.2449	1151.2598, 863.1930, 711.1415, 575.1234, 423.0737, 287.0567	A-type procyanidin tetramer	0.6	0.08±0.00
28	38.57	231.0, 278.2	1150	1149.2306	1149.2317	1149.2452, 575.1214, 407.0796, 285.0403	A-type procyanidin tetramer	0.9	0.01±0.00
29	41.37	232.2, 279.4	578	577.1352	577.1349	577.1392, 451.1053, 425.0888, 407.0784, 289.0717	B-type procyanidin dimer	-0.4	0.73±0.01
30	42.02	235.7, 278.2	576	575.1195	575.1189	449.0889, 423.0727, 285.0401	Procyanidin A2	-1.0	0.63±0.07
31	43.92	231.0, 279.4	864	863.1829	863.1846	863.1916, 711.1418, 575.1217, 449.0888, 411.0728, 289.0713	A-type procyanidin trimer	2.0	0.07±0.00
32	45.63	227.4, 279.4	576	575.1195	575.1193	575.1221, 449.0887, 423.0726, 285.0403, 289.0713	A-type procyanidin dimer	-0.3	N.D

N.D.: Not detected

Compound 1, 2, 4 and 30 were further identified according to the chemical standards. Individual procyanidins were calculated as mg procyanidin B2 equivalent (mg PB2E/100 g DW).

For peaks 2 in F6, the [M-H] ^−^ ion at *m/z* 577.1352 suggested the molecular weight of 578 of a procyanidin dimer with a B-type interflavanoid linkage (Fig. 4C and Table 3) [17]. Amongst the ion products of MS^2^, the ion at *m/z* 451.1054 [M-H-126] ^−^ resulted from the elimination of a phloroglucinol molecular from the B-type dimer, ions at *m/z* 407.0779 [M-H-170]^−^ and *m/z* 289.0716 [M-H-288] ^−^ were from the RDA fission of a B-type dimmer (Table 3) [41]. It was identified as procyanidin B2 according to the literature and chemical standards [41].

For peaks 3 and 4 in F7, the [M-H] ^−^ ion at *m/z* 865.1985 suggested the molecular weight of 866 of a procyanidin trimer with B-type interflavanoid linkage (Fig. 4D and Table 3) [41]. Amongst the ion products of MS^2^ of peaks 3 and 4, the ion at *m/z* 739.1730 and 739.1722 [M-H-126]^−^ resulted from the elimination of a phloroglucinol molecule from the B-type trimer, and the ion at *m/z* 577.1383 and 577.1378 [M-H-288] ^−^ was from the cleavage of the B-type trimer, and ions at *m/z* 407.0781, 407.0793 and 287.0564, 287.0575 were derived from RDA of a B-type trimer (Table 3) [42]. According to the chemical standard confirmation, peak 4 was identified as procyanidin C1, which was identified in the litchi pulp for the first time. For peak 5 in F7, the [M-H] ^−^ ion at *m/z* 863.1892 in peak 5 suggested the molecular weight of 864 of a procyanidin trimer with an A-type interflavanoid linkage (Fig. 4D and Table 3). Amongst the ion products of MS^2^ of peak 5, ions at *m/z* 711.1418 [M-H-152] ^−^, *m/z* 693.1312 [M-H-152-18]^−^ resulted from the RDA fission [42].

In F8, the [M-H] ^−^ ion at *m/z* 575.1195 (peaks 30, 32) and 577.1352 (peaks 12, 29) indicated the presence of both A and B type procyanidin dimmers with molecular weight of 576 and 578, respectively (Fig. 4E and Table 3). Furthermore, compound in peak 30 was identified as procyanidin A2 according to the standard (Table 3).

The [M-H] ^−^ ion at *m/z* 863.1829, 863.1892 (peaks 14, 15, 16, 24, 31) and 865.1987, 865.1985, 865.2019 (peaks 10, 11, 17, 21, 22, 25) indicated the presence of A and B type procyanidin trimers with molecular weight of 864 and 866, respectively (Fig. 4E and Table 3).

The [M-H] ^−^ ion at *m/z* 1149.2306 (peak 28),suggested the molecular weight of 1150 of a procyanidin tetramer with two A-type interflavanoid linkage and one B-type interflavanoid linkage. Amongst the fragments of its MS^2^, the ion at *m/z* 575.1214 [M-H-574] ^−^ and 407.0796 [M-H-168] ^−^ were from the cleavage of the B-type interflavanoid linkage and the RDA of a A-type dimer (Fig. 4E and Table 3). The [M-H] ^−^ ion at *m/z* 1151.2463 (peaks 6, 7, 18, 26) and 1151.2440 (peak 27) suggested the molecular weight of 1152 of procyanidin tetramers with one A-type interflavanoid linkage and two B-type interflavanoid linkage. Due to different positions of A-type linkages in the tetramers, different MS^2^ ions were produced (Fig. 4E and Table 3), which were typical ions of procyanidin oligomer [36]. The [M-H] ^−^ ion at *m/z* 1153.2619 (peaks 19, 20, 23) and 1153.2679 (peak 13) suggested the molecular weight of 1154 of procyanidin tetramers with B-type interflavanoid linkage (Fig. 4E and Table 3). The [M-H] ^−^ ion at *m/z* 1439.3097 (peaks 8, 9) suggested the molecular weight of 1440 of procyanidin pentamers with one A-type interflavanoid linkage. Due to the limit of the ESI scanning range, the MS^2^ ions was not detected for these two pentamers. However, they were confirmed to be procyanidin pentamers according to their UV specture (279.4 nm) and the literature [36].

So far, no procyanidin hexamer was identified in HPCE by LC-ESI-Q-TOF-MS, although [M-H] ^−^ ion signal at *m/z* 1729 was detected in direct infusion ESI-MS (Fig. 2). This may be due to the low concentration of hexamer in the fractions of HPCE.

### Quantification of individual procyanidin in HPCE by LC-ESI-MS

Results of quantification of individual procyanidin showed that (–)-epicatechin (9.88 ± 0.91 mg PB2E/100 g DW), procyanidin B2 (12.25 ± 0.71 mg /100 g DW), procyanidin C1 (1.62 ± 0.11 mg PB2E/100 g DW) and A-type procyanidin trimer (1.21 ± 0.11 mg PB2E/100 g DW) accounted for 84.93% (W/W) of procyanidins identified in HPCE (Table 3). For other cultivars, the monomer (–)-epicatechin and dimer procyanidin B2 were also two main procyanidins in the litchi pulp, but with much lower concentrations when compared with those of HPCE (data not shown). In a recent study investigating the oligomeric proanthocyanidins in plant products such as grape seed extracts by using UHPLC-PDA-ESI/HRMS^n^, proanthocyanidins accounted for 48.79% of the grape seed extract (w/w on dry weight basis), among which mononers such as catechin, epicatechin, epicatechin-gallate, and catechin-gallate accounted for 16.63%, dimmers accounted for 17.44%, trimer accounted for 14.24% of the extract [3]. In lowbush blueberry, red apple and mangos, the oligomeric proanthocyanidins (DP ⩽ 3) accounted for 12.42%, 25.63% and 44.88% of the total proanthocyanidins (w/w), respectively [43].

### Antioxidant activity assay

**Fig 5:**
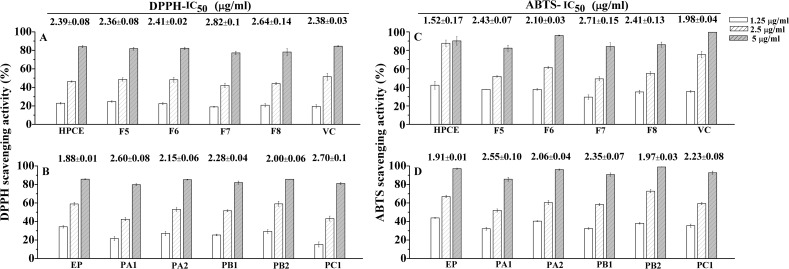
Antioxidant activity of HPCE, fractions, and procyanidins standards by DPPH (A, B) and ABTS (C, D) assays. EP stands for (–)-epicatechin, and PA1, PA2, PB1, PB2, PC1 stand for the standards of procyanidin A1, A2, B1, B2, and C1, respectively.

DPPH and ABTS assays were commonly used to evaluate antioxidant activity of various phenolic extracts *in vitro*. In the present study, the radical scavenging activities of different fractions of HPCE as well as six procyanidins standards and Vc were evaluated by both DPPH and ABTS assays (Fig. 5). As far as their IC_50_ were concerned, the HPCE and its different fractions showed similar antioxidant activity with that of Vc (2.38 ± 0.03 μg/ml for DPPH assay and 1.98 ± 0.04 μg/ml for ABTS assay). Further analysis of six individual procyanidin standards also showed similar radical scavenging capacities, the IC_50_ of which ranged from 1.88 ± 0.01 μg/ml (EP) to 2.70 ± 0.10 μg/ml (PC1) for DPPH assay, and from 1.91 ± 0.01 μg/ml (EP) to 2.55 ± 0.10 μg/ml (PA1) for ABTS assay. Such results showed that the procyanidins identified in HPCE may contribute the antioxidant activities of the litchi pulp extracts.

## Conclusion

The contents of total proanthocyanidins as well as total phenolics, flavonoids, and antioxidant activities of the pulp of 32 litchi cultivars were evaluated in the present study. Hemaoli showed the highest proanthocyanidins content and antioxidant activities. ESI-MS and NMR analysis demonstrated that the procyandins composed of (*epi*)catechin unites with DPs of 2–6 were dominant proanthocyanidins in HPCE. By using LC-ESI-Q-TOF-MS, 32 procyanidins was identified in litchi pulp for the first time. Quantification of individual procyanidin in HPCE indicated that (–)-epicatechin, procyanidin B2, procyanidin C1 and A-type procyanidin trimer were the majority of main procyanidins in litchi pulp. HPCE fractions and six individual procyanidins all had high radical scavenging activities as shown by DPPH and ABTS assays. Therefore, litchi cultivars rich in proanthocyanidins are good resources of dietary antioxidants and may have health-promoting benefit to human health. As far as the potential health benefits of proanthocyanidins are concerned, results of the present study may play important role in litchi breeding program as well as the development of litchi fruit industry.

## Supporting Information

S1 Table Harvest places and time of 32 litchi cultivars. (PDF)
