# Supplementary material for: Identification of Proanthocyanidins from Litchi (Litchi chinensis Sonn.) Pulp by LC-ESI-Q-TOF-MS and Their Antioxidant Activity
Source: PLoS One. 2015 Mar 20;10(3):e0120480. doi: 10.1371/journal.pone.0120480 (PMC4368102; doi:10.1371/journal.pone.0120480)
Supplement: S1 Table — (PDF) [file pone.0120480.s001.pdf]

Table S1. Harvest places and time of 32 litchi cultivars.

No.	Cultivar	Harvest place	Harvest time	No.	Cultivar	Location	Harvest time
1	Baila	Maoming, Guangdong	2011. 6. 16	17	Jinganghongnuo	Conghua, Guangdong	2011. 7. 18
2	Baitangying	Haikou, Hainan	2011. 5. 30	18	Jizuili	Lianjiang, Guangdong	2011. 7. 08
3	Caomeili	Qinzhou, Guangxi	2011. 7. 14	19	Lanzhu	Zhangzhou, Fujian	2011. 7. 18
4	Chenzi	Putian, Fujian	2011. 7. 31	20	Lingfengnuo	Dongguan, Guangdong	2011. 7. 08
5	Dadingxiang	Haikou, Hainan	2011. 6. 27	21	Miaozhongnuo	Guangzhou, Guangdong	2011. 7. 06
6	Dazao	Maoming, Guangdong	2011. 6. 22	22	Mili	Qinzhou, Guangxi	2011. 7. 08
7	Feizixiao	Hailou, Hainan	2011. 5. 30	23	Nuomici	Guangzhou, Guangdong	2011. 7. 06
8	Guifeihong	Qinzhou, Guangxi	2011. 7. 20	24	Qinzhouhongli	Qinzhou, Guangxi	2011. 6. 30
9	Guiwei	Dongguan, Guangdong	2011. 7. 08	25	Sanyuehong	Qinzhou, Guangxi	2011. 6. 08
10	Hemaoli	Yuanyang, Yunnan	2011. 6. 01	26	Shuangjianyuhebao	Yangxi, Guangdong	2011. 7. 14
11	Heiye	Maoming, Guangdong	2011. 6. 16	27	Shuidong	Guangzhou, Guangdong	2011. 6. 08
12	Hexiachuan	Maoming, Guangdong	2011. 6. 22	28	Wuheli	Haikou, Hainan	2011. 6. 27
13	Huaizhi	Guangzhou, Guangdong	2011. 7. 06	29	Xianpoguo	Guangzhou, Guangdong	2011. 7. 06
14	Hongxiuqiu	Dongguan, Guangdong	2011. 7. 08	30	Yingshanhong	Qinzhou, Guangxi	2011. 7. 20
15	Jianjianghongnuo	Maoming, Guangdong	2011. 7. 08	31	Yuanhong	Ningde, Fujian	2011. 7. 31
16	Jinfeng	Maoming, Guangdong	2011. 6. 30	32	Ziniangxi	Haikou, Hainan	2011. 6. 27
